# Albumin-to-Fibrinogen Ratio Independently Predicts 28-Day Mortality in Patients with Peritonitis-Induced Sepsis

**DOI:** 10.1155/2020/7280708

**Published:** 2020-05-06

**Authors:** Huiyu Tai, Zhiyun Zhu, Haifeng Mei, Wenbin Sun, Wei Zhang

**Affiliations:** ^1^Department of Intensive Care Unit, Taizhou People's Hospital, Medical School of Nantong University, China; ^2^Department of Infectious Disease, Taizhou People's Hospital, Medical School of Nantong University, China

## Abstract

**Background:**

This study is aimed at investigating whether albumin-to-fibrinogen ratio (AFR) could independently predict the prognosis in patients with peritonitis-induced sepsis.

**Methods:**

A total of 246 eligible patients who were scheduled to undergo surgical treatment for peritonitis-induced sepsis were enrolled in this study. The primary observational endpoint was 28-day hospital mortality. Cox proportional hazards regression analysis with the Wald test was performed to identify prognostic factors for 28-day mortality in septic patients. Receiver operating characteristic (ROC) and Kaplan-Meier curve analyses were carried out to evaluate the association of baseline AFR and prognosis in septic patients.

**Results:**

Of all the cohort study participants, there were 59 nonsurvivors with a 28-day mortality of 24.0% (59/246). Baseline AFR (hazard ratio (HR): 0.67, 95% confidence interval (CI): 0.42–0.93, *P* = 0.018) and the presence of septic shock (HR: 2.43, 95% CI: 1.42–3.91, *P* = 0.021) were two independent prognostic factors for 28-day mortality in patients with peritonitis-induced sepsis by multivariate Cox analysis. Baseline AFR was a significant predictor for 28-day mortality with an area under the curve (AUC) of 0.751, a cut-off value of 8.85, a sensitivity of 66.10%, and a specificity of 70.05%, respectively (95% CI: 0.688–0.813, *P* < 0.001). A low baseline AFR level (≤8.85) was significantly associated with a lower overall survival rate in septic patients by Kaplan-Meier curve analysis with log-rank test (*P* = 0.004).

**Conclusions:**

This study indicates that AFR independently predicts 28-day mortality in patients with peritonitis-induced sepsis.

## 1. Introduction

As one of the most common causes of postoperative death, the incidence of sepsis was increasing, the same as organ dysfunction [1]. Despite advances in the pathophysiology understanding and therapeutic strategy improvement, the mortality rate caused by severe sepsis or septic shock still remains high [2]. Furthermore, Liu et al. have reported that the abdomen and pulmonary infection occupy the most frequent etiologies of severe sepsis or septic shock [3]. The sepsis-induced mortality rate is reported to be very high, ranging from 20% to 30% [4, 5]. As a result, early effective risk stratification and timely management are critically needed for the outcome improvement in patients with sepsis.

Albumin (Alb), a well-established traditional nutritional and inflammatory biomarker, is shown to be a prognostic biomarker in patients with sepsis [5]. Fibrinogen (Fib), another common inflammatory protein, plays a key role in the coagulation cascade and it is closely associated with tumor development [6]. Alb-to-Fib ratio (AFR), consisting of Alb and Fib, is shown to be an effective biomarker reflecting nutritional and coagulation status, as well as the inflammatory condition. However, whether AFR could act as a prognostic factor for patients with peritonitis-induced sepsis remains unclear. This study is aimed at investigating potential prognostic factors including AFR for septic patients.

## 2. Material and Methods

### 2.1. Patients

This retrospective observational study was approved by the Medical Institutional Ethics Committee of Taizhou People's Hospital, Medical School of Nantong University. Eligible patients who were scheduled to undergo surgical treatment for peritonitis-induced sepsis between May 2015 and May 2018 were enrolled in this study. Inclusion criteria are as follows: (1) adult patients aged over 18 years with both gender; (2) presence of sepsis according to the definition criteria [7] induced by acute peritonitis; and (3) admitted to the intensive care unit (ICU) after emergency abdominal surgery. Those patients aged <18 years, with pregnancy, hematologic diseases, hepatic dysfunction, sepsis induced by infections in other sites, and who received glucocorticoid or other immunosuppressant treatment were excluded. Those patients without complete 28-day follow-up data were also excluded.

### 2.2. Data Collection

The data were collected from medical records of the enrolled patients. The demographics including age, gender, and body mass index (BMI); baseline clinical characteristics including active smoking habits, history of previous abdominal surgery, blood culture results, mean atrial pressure, body temperature, heart rate, respiratory rate, and duration of operation; and the intervention strategies including mechanical ventilation, renal replacement therapy, and norepinephrine therapy were recorded in detail. Preoperative comorbidities including hypertension, diabetes mellitus, cardiac disease, chronic renal disease, chronic lung disease, malignancy, and cerebrovascular disease were also retrieved from the database. In order to assess the disease severity, American Society of Anesthesiologists (ASA) physical status, Acute Physiology and Chronic Health Evaluation (APACHE) II score, Sepsis-related Organ Failure Assessment (SOFA) score [8], Simplified Acute Physiology Score (SAPS) III [9], and modified Charlson comorbidity index (MCCI) [10] were also calculated according to the methods by the previous literatures.

### 2.3. Endpoint

The patients were admitted to the intensive care unit (ICU) postoperatively and managed according to the international guidelines for severe sepsis and septic shock [11]. The primary observational endpoint was 28-day hospital mortality. As for those patients who were discharged within 28 days, the follow-up was carried out using a structured telephone. The second observational endpoint was 12 months after the surgery. Patients were followed up via telephone and outpatient interview. The life quality of enrolled patients at two time points (3 months and 12 months) was evaluated using three scales, the Euroqol-5 dimension (EQ-5D), the Mos 36-item Short Form Health Survey (SF-36), and the Activities of Daily Living (ADL) [12].

### 2.4. Nutritional Status Assessment among Survived Septic Patients

Nutritional status assessment indexes including prognostic nutritional index (PNI) and nutrition risk screening (NRS) 2002 [13] were evaluated at 3 months and 12 months after the surgery. As described by previous reports, the PNI was calculated by the following formula: 10 × albumin value (g/dL) + 0.005 × total lymphocyte count [14].

### 2.5. Laboratory Tests

In all enrolled cases, peripheral venous blood samples were obtained in the early morning on the surgery day. The complete blood counts including white blood cell (WBC), hemoglobin, lymphocyte, and platelet count and blood chemistry including Alb, creatinine, and Fib and arterial blood gas analysis including lactic acid and potential of hydrogen (pH) were measured and routinely recorded at our institution. Preoperative serum inflammatory cytokines including C-reactive protein (CRP) and procalcitonin (PCT) were also detected using the methods of enzyme-linked immunosorbent assay (ELISA) with antibodies (R&D Systems, CA, USA).

### 2.6. Statistical Analysis

Statistical analyses were performed using the software of SPSS 23.0 (SPSS, Inc., IA, USA) and GraphPad Prism 8.0 (GraphPad Inc., CA, USA). Continuous variables are presented as the mean ± standard error (SE) and categorical variables as number with percentages. The chi-square test, Fisher exact test, Mann–Whitney *U* test, and Student test were used for data analysis as appropriate. Cox proportional hazards regression analysis with the Wald test was performed to identify prognostic factors for 28-day mortality in septic patients. Receiver operating characteristic (ROC) curve analysis was carried out to evaluate the predictive role of baseline AFR for 28-day mortality. Furthermore, Kaplan-Meier curve analysis was performed to assess the association between baseline AFR and survival rate. A two-sided *P* value < 0.05 was considered statistically significant.

## 3. Results

### 3.1. Patient Characteristics

During the 3-year study period, 286 consecutive patients with peritonitis-induced sepsis were initially enrolled. Of these patients, 40 were excluded basing on the exclusion criteria (10 with hematologic diseases, 12 with hepatic dysfunction, 10 received glucocorticoid or other immunosuppressant treatment, and 8 lost to follow-up). As a result, 246 patients were finally included in the analysis. The mean age of the cohort was 69.1 years, and the majority (154/246, 62.6%) was male patients. Of all the cohort study participants, there were 59 nonsurvivors with a 28-day mortality of 24.0% (59/246). Baseline characteristics of survivors and nonsurvivors are summarized in Table 1 with details. No significant differences were observed with respect to age, gender distribution, BMI, active smoking habits, history of previous abdominal surgery, and duration of operation. Baseline comorbidities between two groups were not statistically different except hypertension and chronic renal disease. The nonsurvivors had higher baseline APACHE II, SOFA, and SAPS III scores in comparison with survivors. The vital signs including mean atrial pressure, body temperature, heart rate, and respiratory rate did not differ considerably between two groups. Moreover, those patients with septic shock and who received mechanical ventilation, renal replacement therapy, and norepinephrine therapy were related to an increased 28-day mortality rate.

### 3.2. Laboratory Tests

Furthermore, we compared laboratory variables between survivors and nonsurvivors. As indicated in Table 2, the results indicated that nonsurvivors had higher levels of lactic acid, CRP, and PCT than survivors. In addition, patients showed a significantly lower baseline AFR level in nonsurvivors when compared with survivors.

### 3.3. Prognostic Factors for 28-Day Mortality

To investigate potential prognostic factors for 28-day mortality in septic patients, univariate and multivariate Cox proportional hazards regression analyses were performed using the thirteen potential risk factors (*P* < 0.05 in Tables 1 and 2). The univariate Cox analysis indicated that the presence of septic shock, baselines levels of SOFA, SAPS III score, lactic acid, CRP, and AFR were six potential prognostic factors for septic patients (see Table 3). All variables with a *P* value < 0.1 in univariate analyses were eligibly included in the multivariate analysis. Baseline AFR and the presence of septic shock were two independent prognostic factors for 28-day mortality in patients with peritonitis-induced sepsis.

### 3.4. Baseline AFR and Prognosis

**Figure 1:**
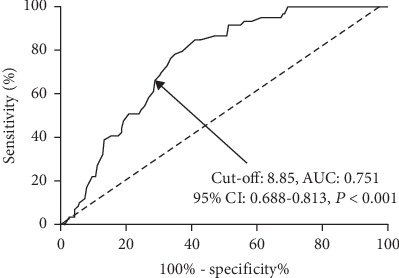
The predicative value of baseline AFR for 28-day mortality in septic patients by ROC analysis. AUC: 0.751, cut-off value: 8.85, sensitivity: 66.10%, specificity: 70.05%, *P* < 0.001. AFR: albumin-to-fibrinogen ratio; ROC: receiver operating characteristics; AUC: area under the ROC curve.

**Figure 2:**
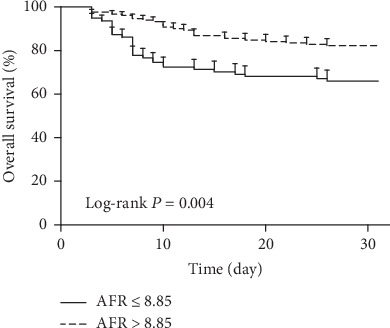
The overall survival rate and baseline AFR by Kaplan-Meier curve analysis. A low baseline AFR level (≤8.85) was significantly associated with a lower overall survival rate in septic patients by log-rank test (*P* = 0.004). AFR: albumin-to-fibrinogen ratio.

As shown in Figure 1, baseline AFR was a significant predictor for 28-day mortality with an area under the curve (AUC) of 0.751, a cut-off value of 8.85, a sensitivity of 66.10%, and a specificity of 70.05%, respectively. According to the cut-off value, patients were categorized into high AFR (>8.85) and low AFR (≤8.85) groups.  Figure 2 shows that a low baseline AFR level was significantly associated with a lower overall survival rate in septic patients by Kaplan-Meier curve analysis with log-rank test.

### 3.5. Follow-Ups of Survived Septic Patients

Among the 187 survivors within 28 days after the surgery, only 122 survived patients finished the 12-month follow-up (13 died, 25 out of touch, and 27 rejected to cooperate). The results of life quality and nutritional status-associated variables among survived septic patients at 3 months and 12 months are summarized in Table 4. The survivors had significantly higher scores of physical functioning, bodily pain, general health, health utility, and activities of daily living at 12 months when compared with those at 3 months after surgery (*P* < 0.05), indicating the improved life quality along with time. Moreover, patients at 12 months had lower NRS 2002 and higher PNI scores than at 3 months (*P* < 0.05), suggesting the improved nutritional status.

## 4. Discussion

This retrospective observational study demonstrated that the presence of septic shock and a low baseline AFR level were two independent predictors for 28-day mortality in patients with peritonitis-induced sepsis. Therefore, AFR calculation might be useful to identify septic patients with a high risk of 28-day mortality. To the best of our knowledge, our study firstly indicated baseline AFR level as a prognostic factor in patients with peritonitis-induced sepsis.

Sepsis, which is described as an infection-induced systemic inflammatory response, is a common disorder with a high morbidity and mortality, especially for the older patients [1]. However, prognostic factors for elderly septic patients have not been clearly identified despite the poor prognosis of sepsis in aging patients. Our results from the univariate Cox analysis suggested that the presence of septic shock, baselines levels of the SOFA, SAPS III score, lactic acid, CRP, and AFR were six potential prognostic factors for septic patients. The SOFA score method has already been validated and is currently widely used among septic patients in the ICU [15]. However, a systematic review by Calle et al. concluded that the SOFA score could not be accurately used in the hospital outside the ICU [16]. A recent study by Jahn et al. showed that SAPS III score exhibited high accuracy in prognosis prediction in patients with internal disorders [17]. Another pilot study by Manu et al. also suggested the SAPS III score as a robust prognostic parameter for septic patients [18]. However, some other studies did not support the prognostic role of the SAPS score in septic patients [19]. Our results were not at all consistent with these reports of sepsis. Further investigations may be necessary to validate the prognostic role of SOFA and SAPS III score for septic patients. As a consequence of tissue hypoxia, increased blood lactate concentration is very common in septic patients and it is closely associated with increased morbidity and mortality [20]. In normotensive patients with sepsis, a high lactate concentration (>4 mmol/L) independently predicted a higher mortality [21]. CRP, as an acute phase reactive protein, was reported to be able to evaluate the prognosis of elderly patients with pulmonary infection-induced sepsis with controversial results [22]. However, our multivariate Cox proportional hazards regression analysis only supported septic shock and baseline AFR as independent prognostic factors for peritonitis-induced sepsis. In our consideration, the different cohort characteristics, age ranges, infection sites, intervention strategies, and observational indexes might be the potential explanations for the different conclusions. Septic shock is a well-established factor associated with a poor prognosis in septic patients [23], which is in support with our results.

Alb is commonly utilized as a sensitive and effective biomarker inflecting the nutritional and inflammatory status [24]. Expressions of Alb effectively reflect the organic function, nutritional status, and prior physical activity of patients [25]. Decreased serum Alb expressions were widely observed in septic patients. In addition, hypoalbuminemia can significantly alter the pharmacokinetics of antimicrobials and low Alb levels impair antimicrobial expressions in septic patients. This may be a potential hypothesis for the prognostic role of albumin in sepsis [25]. Fib is a key protein in the blood coagulation cascade as well as an acute phase reaction protein for systemic inflammation like CRP [6]. Increased serum Fib expression is observed in patients with sepsis [26]. Fib is an acute phase reactant, and its consumption indicates the activation of coagulation in patients with acute infection. Dysregulated circulating Fib expressions in septic patients were closely associated with the development of disseminated intravascular coagulation (DIC), which is well known to be a poor prognostic factor [27]. AFR, a novel immune biomarker which takes Alb and Fib into account, has been widely proposed as a prognostic marker in various diseases. AFR was stated to be a superior prognostic biomarker in non-small-cell lung cancer (NSCLC) individuals when comparing with Alb and Fib [28]. A recent study by Ying et al. indicated pretreatment AFR as a promising predictor for the survival of advanced NSCLC patients after first-line platinum-based chemotherapy [29]. Moreover, another study by Yu et al. has also reported the prognostic role of preoperative AFR for patients with advanced epithelial ovarian cancer [30]. In our minds, AFR could effectively reflect the status of nutrition, inflammation, and coagulation function in septic patients. Furthermore, the status of nutrition, inflammation, or coagulation is closely linked to the disease severity and prognosis in septic patients. Furthermore, systemic inflammation may probably be involved in the pathogenesis and may be the leading cause of death in sepsis [31]. These might be possible explanations for the prognostic role of baseline AFR in patients with peritonitis-induced sepsis.

This study has some limitations. First, this is a single-center study with a retrospective nature and a relatively small sample size. Second, the involved mechanisms remain unknown. Third, some confounding factors which can impact the expressions of Alb or Fib (e.g., nutritional status and operation stimulation) may affect the conclusions. Last, whether approaches to improve baseline AFR levels can significantly improve clinical outcomes remains unclear.

## 5. Conclusions

In conclusion, our study indicated that the presence of septic shock and baseline AFR level were two independent prognostic factors for 28-day mortality in patients with peritonitis-induced sepsis.

## Figures and Tables

**Table 1:** Baseline characteristics in septic patients.

Parameters	Total (*n* = 246)	Survivors (*n* = 187)	Nonsurvivors (*n* = 59)	*P* value
Age (years)	69 (52-82)	69 (55-82)	70 (52-79)	0.31
Gender, *n* (%)	—	—	—	0.34
Male	154 (62.6)	114 (61.0)	40 (67.8)	—
Female	92 (37.4)	73 (39.0)	19 (32.2)	—
BMI (kg/m^2^)	21.9 ± 2.0	21.9 ± 1.8	22.0 ± 2.1	0.72
Comorbidities, *n* (%)	—	—	—	—
Hypertension	67 (27.2)	44 (23.5)	23 (39.0)	0.020^∗^
Diabetes mellitus	48 (19.5)	34 (18.2)	14 (23.7)	0.35
Cardiac disease	25 (10.2)	19 (10.2)	6 (10.2)	0.99
Chronic renal disease	25 (10.2)	15 (8.0)	10 (5.3)	0.048^∗^
Chronic lung disease	23 (9.3)	16 (8.6)	7 (11.9)	0.45
Malignancy	27 (11.0)	20 (10.7)	7 (11.9)	0.80
Cerebrovascular disease	32 (13.0)	24 (12.8)	8 (13.6)	0.89
Active smoking habits, *n* (%)	45 (18.3)	35 (18.7)	10 (16.9)	0.76
Previous abdominal surgery, *n* (%)	89 (36.2)	72 (38.5)	17 (28.8)	0.18
APACHE II score	19.1 ± 4.5	18.7 ± 4.3	20.2 ± 4.7	0.023^∗^
SOFA score	7.8 ± 1.9	7.5 ± 1.5	9.0 ± 1.7	<0.001^∗^
SAPS III score	67.7 ± 9.2	66.9 ± 8.8	70.4 ± 9.3	0.009^∗^
MCCI	2.0 ± 0.6	2.0 ± 0.6	2.1 ± 0.5	0.25
ASA physical status	2.6 ± 0.8	2.6 ± 0.7	2.8 ± 0.8	0.066
Septic shock, *n* (%)	91 (37.0)	54 (28.9)	37 (62.7)	<0.001^∗^
Mean atrial pressure (mmHg)	68.4 ± 9.8	68.3 ± 10.1	68.7 ± 9.5	0.79
Body temperature (°C)	37.4 ± 0.5	37.4 ± 0.4	37.5 ± 0.6	0.14
Heart rate (per minute)	108.7 ± 8.8	108.2 ± 8.1	110.3 ± 9.3	0.095
Respiratory rate (per minute)	20.1 ± 0.9	20.1 ± 0.9	20.3 ± 0.8	0.13
Duration of operation (min)	138.9 ± 25.8	138.4 ± 24.1	140.4 ± 29.3	0.60
Mechanical ventilation, *n* (%)	182 (74.0)	128 (68.4)	54 (91.5)	<0.001^∗^
Renal replacement therapy, *n* (%)	30 (12.2)	18 (9.6)	12 (20.3)	0.028^∗^
Norepinephrine therapy, *n* (%)	63 (25.6)	27 (14.4)	36 (61.0)	<0.001^∗^
Positive blood culture, *n* (%)	93 (37.8)	69 (36.9)	24 (40.7)	0.60

BMI: body mass index; APACHE: Acute Physiology and Chronic Health Evaluation; SOFA: Sepsis-related Organ Failure Assessment; SAPS: Simplified Acute Physiology Score; MCCI: modified Charlson comorbidity index; ASA: American Society of Anesthesiologists. *P* values were calculated by chi-square test, Fisher exact test, Mann–Whitney *U*, or *t* test. ^∗^*P* < 0.05.

**Table 2:** Laboratory tests in septic patients.

Parameters	Total (*n* = 246)	Survivors (*n* = 187)	Nonsurvivors (*n* = 59)	*P* value
WBC (10^9^/L)	12.5 ± 3.6	12.4 ± 3.1	13.0 ± 3.8	0.22
Hemoglobin (g/dL)	10.4 ± 1.1	10.3 ± 1.0	10.6 ± 1.2	0.057
Platelet count (10^9^/L)	134.6 ± 36.3	133.1 ± 33.2	139.3 ± 41.3	0.24
Lactic acid (mmol/L)	3.1 ± 0.9	2.9 ± 0.8	3.5 ± 1.1	<0.001^∗^
pH	7.32 ± 0.09	7.32 ± 0.08	7.30 ± 0.09	0.11
CRP (mg/L)	36.7 ± 17.5	33.6 ± 12.4	46.6 ± 22.5	<0.001^∗^
PCT (*μ*g/L)	14.0 ± 8.1	13.3 ± 6.7	16.1 ± 9.1	0.011^∗^
Creatinine (mg/dL)	1.6 ± 0.4	1.6 ± 0.4	1.5 ± 0.4	0.095
AFR	9.4 ± 1.8	9.8 ± 1.9	8.3 ± 1.5	<0.001^∗^

WBC: white blood cell; pH: potential of hydrogen; CRP: C-reactive protein; PCT: procalcitonin; AFR: albumin-to-fibrinogen ratio. *P* values were calculated by Mann–Whitney *U* or *t* test. ^∗^*P* < 0.05.

**Table 3:** Risk factors for 28-day mortality in septic patients by univariate and multivariate Cox proportional hazards regression analysis.

Variables	Univariate		Multivariate	
	HR (95% CI)	*P* value	HR (95% CI)	*P* value
Age (≥69 vs. <69)	1.74 (0.54-5.39)	0.365		
Hypertension (yes vs. no)	1.01 (0.97–1.06)	0.71		
Chronic renal disease (yes vs. no)	1.19 (0.71–1.91)	0.49		
APACHE II score (high vs. low)	1.37 (0.90–2.01)	0.17		
SOFA score (high vs. low)	1.35 (1.01–1.91)	0.038^∗^	1.39 (0.86–2.19)	0.12
SAPS III score (high vs. low)	1.65 (1.18–2.27)	0.017^∗^	1.62 (0.66–3.63)	0.27
Septic shock (yes vs. no)	2.31 (1.26–4.47)	0.011^∗^	2.43 (1.42–3.91)	0.021^∗^
Mechanical ventilation (yes vs. no)	1.63 (0.65–3.77)	0.28		
Renal replacement therapy (yes vs. no)	2.67 (0.80–8.33)	0.19		
Norepinephrine therapy (yes vs. no)	1.01 (0.95–1.07)	0.48		
Lactic acid (high vs. low)	1.09 (1.01–1.26)	0.022^∗^	1.72 (0.96–3.05)	0.09
CRP (high vs. low)	1.31 (1.05–1.62)	0.013^∗^	1.37 (0.75–2.42)	0.24
PCT (high vs. low)	1.40 (0.66–2.96)	0.37		
AFR (high vs. low)	0.84 (0.75–0.94)	0.009^∗^	0.67 (0.42–0.93)	0.018^∗^

APACHE: Acute Physiology and Chronic Health Evaluation; SOFA: Sepsis-related Organ Failure Assessment; SAPS: Simplified Acute Physiology Score; CRP: C-reactive protein; PCT: procalcitonin; AFR: albumin-to-fibrinogen ratio; HR: hazard ratio; CI: confidence interval. ^∗^*P* < 0.05.

**Table 4:** The follow-ups of survived septic patients.

Parameters	3 months (*n* = 122)	12 months (*n* = 122)	*P* value
Physical functioning	65.4 ± 10.3	68.7 ± 9.3	0.009^∗^
Role physical	59.3 ± 11.4	60.0 ± 12.3	0.645
Bodily pain	66.7 ± 8.9	69.7 ± 10.8	0.019^∗^
Vitality	65.8 ± 8.7	66.3 ± 9.8	0.674
General health	55.9 ± 10.1	59.3 ± 8.8	0.006^∗^
Social functioning	66.5 ± 9.3	67.4 ± 10.3	0.474
Role emotional	73.4 ± 12.5	75.1 ± 13.3	0.305
Health utility scores	0.90 ± 0.05	0.95 ± 0.02	<0.001^∗^
Mental health	71.8 ± 8.7	72.6 ± 9.1	0.483
Activities of daily living	97.1 ± 0.8	99.0 ± 0.3	<0.001^∗^
NRS 2002	2.5 ± 1.1	2.1 ± 1.2	0.007^∗^
PNI	42.4 ± 7.2	46.3 ± 8.7	<0.001^∗^

NRS: nutrition risk screening; PNI: prognostic nutritional index. ^∗^*P* < 0.05.

## Data Availability

Please contact the corresponding author (Wei Zhang, zhangweitz@hotmail.com) for data requests.
